# Breaking embolic ground: the clot thickens in intermediate-risk pulmonary embolism

**DOI:** 10.1093/ehjopen/oeaf099

**Published:** 2025-08-12

**Authors:** Philippe Guerci, Romain Chopard

**Affiliations:** Department of CardioThoracic and Vascular Anesthesiology and Critical Care Medicine, University Hospital of Nancy, Nancy, France; INSERM U1116, DCAC, University of Lorraine, Nancy, France; Department of Cardiology, Centre Hospitalier Universitaire de Besançon, Besançon, France; SINERGIES Laboratory, University Marie & Louis Pasteur, Besançon, France; F-CRIN, INNOVTE Network, Saint-Étienne, France

**This editorial refers to ‘Endovascular management of intermediate-risk pulmonary embolism: evidence, outstanding questions, drivers of utilization, and the horizon’, by A.A. Shahriar *et al*., https://doi.org/10.1093/ehjopen/oeaf071.**

Over the past decade, pulmonary embolism (PE) management has entered a transformative era, particularly for the broad and clinically heterogeneous group of intermediate-risk patients. While systemic anticoagulation remains the cornerstone of therapy as suggested by most guidelines,^1,2^ a surge in the use of endovascular interventions—despite a still-evolving evidence base—signals both an innovation opportunity and a cautionary tale. Indeed, the procedural armamentarium available has expanded dramatically in recent years. In their timely review, Shahriar *et al*.^3^ provides a comprehensive synthesis of the evolving landscape and calls for an evidence-informed recalibration of practice^3^.

## Evidence lagging behind utilization

Intermediate-risk PE, especially the intermediate-high risk subset characterized by right ventricular (RV) dysfunction and biomarker elevation, accounts for 25–60% of hospitalized PE cases.^4^ While this population carries a non-negligible risk of early decompensation (3–15%), the benefit of full-dose systemic thrombolysis in preventing haemodynamic collapse was offset by an increased risk of major bleeding, including haemorrhagic stroke in the PEITHO trial.^5^ Thus, the unmet need is real, and the desire for an active, interventional solution is understandable. Current guidelines constrain its use to patients who decompensate on anticoagulation alone.^1^ Into this therapeutic void, endovascular therapies promise a middle ground: rapid pulmonary reperfusion with improved safety profiles.

Yet, as underscored by Shahriar *et al*., the expansion of percutaneous thrombectomy and catheter-directed thrombolysis has far outpaced the accumulation of high-quality evidence^3^. To date, five percutaneous devices have received FDA clearance via the 510(k) pathway—EKOS™, FlowTriever®, Indigo®, Bashir™, and AlphaVac™—primarily on the basis of single-arm studies showing short-term improvement in surrogate markers such as the RV/LV ratio or systolic pulmonary arterial pressure. Only three small randomized controlled trials (RCTs) (ULTIMA, Kroupa, CANARY) have compared endovascular therapy against anticoagulation alone,^6–8^ and none were powered for patient-centred outcomes like mortality or symptom burden, and long-term data remain scarce.

## A rising curve with shaky foundations

Despite this evidence gap, procedural use continues to rise. Between 2017 and 2020, up to 20–26% of intermediate-risk patients at Pulmonary Embolism Response Team (PERT) centres received catheter-based interventions.^9^ Several factors may contribute, including the relative ease of use and safety profile, the emergence of PERTs, the promotion of registries and real-world data, or potential financial incentives. While some of these forces are familiar to interventionalists, they warrant scrutiny when the benefit-risk balance remains unproven. Position paper,^10^ as well as the one by Shahriar *et al*., may help physicians navigate current uncertainties and adopt these new devices in a thoughtful and appropriate way, pending the publication of robust data.

## Awaiting the tipping point

Indeed, the field awaits the results of three pivotal trials—HI-PEITHO, PEERLESS II, and PE-TRACT—that will collectively determine whether endovascular therapy improves short- or long-term outcomes in intermediate-high risk PE. These trials vary in design, but all incorporate robust, patient-centred efficacy endpoints such as mortality, decompensation, dyspnoea, or functional recovery.^11–13^ Additionally, the PEITHO-3 trial, testing reduced-dose systemic thrombolysis, may also provide a pharmacological alternative to endovascular options, particularly in low-resource settings.^14^

Should these RCTs demonstrate a net clinical benefit, percutaneous strategies may gain stronger endorsement in guidelines and potentially reshape the acute management of intermediate-high risk PE in the near future. Conversely, if risks outweigh benefits or no significant advantage emerges over anticoagulation alone, the field must be prepared for a medical reversal—a scenario not unfamiliar to cardiology. In either case, these parallel therapeutic explorations underscore the importance of conducting comparative cost-effectiveness analyses and engaging in nuanced shared decision-making with patients.

## Therapeutic inertia?

Current guidelines offer clear directives: anticoagulation remains the standard of care for intermediate-risk PE (Class I), while catheter-based therapies are reserved for cases of clinical deterioration or contraindication to thrombolysis (Class IIa, Level C).^1^ Yet, for interventionalists, rigid adherence to this paradigm—though grounded in caution—risks reinforcing a form of therapeutic inertia that may inadvertently hinder innovation.

The advent of catheter-based strategies in PE represents both a technical milestone and a clinical responsibility. Until definitive trial results are available, selective restraint should not be mistaken for therapeutic inertia. Rather, it reflects a mature commitment to patient-centred, evidence-driven care. The clot may thicken—but so too must the rigour with which we justify every intervention.
